# Letter from Dr. D. P. Gregg, on the Insertion of Artificial Teeth

**Published:** 1858-01

**Authors:** Decatur P. Gregg

**Affiliations:** Greensboro', N. C.


### ARTICLE X.

### *Letter from* DR. D. P. GREGG, *on the Insertion of Artificial Teeth.*

EDITORS OF THE JOURNAL:

*Sirs*—After considerable delay in satisfying my mind of the utility of a somewhat different mode of operating I have adopted in certain cases, I have concluded to call your attention to it. If others have been in the habit of performing it, I have not been fortunate enough to have seen any notice of it in my discursive readings. If others have done it, without calling general attention to it, it only evinces to my mind the necessity of giving it a greater perfection in detail if not in conception. This much for a preface and apology for wishing to occupy your pages.

There are two general modes of inserting teeth, and these two modes are subdivided into several varieties. Those consist in inserting with pivot upon the fang or upon plate without the fangs. It has been the practice long since adopted, to insert with a wooden or metallic pivot a single tooth upon one fang. Many have sought to combine the advantages of inserting them upon plate with a pivot for greater security in retention, without attempting as I conceive to obviate the objections that must always arise from having a plate in the mouth and placing clasps around the teeth. To combine the advantages of both, and avoid some of their evils seems to me to be a desideratum not only in a great measure attainable but an object worthy our constant efforts. How far I have succeeded in this you and the profession must judge. I indulge the hope that I have at least taken one step in that direction.

With these objects in view, and to meet the peculiar exigencies of many cases not otherwise conveniently remediable by art, I have for more than two years adopted a mode of practice that has met my most sanguine expectations.

My method is to introduce a gold tube (see Prof. Harris' work on Dental Surgery) into the fang and filling around the tube with gold foil—thereby making a solid base for the crown of the artificial tooth, I wish to insert. In most cases ordinary gold foil used in thin folds or strips, will be found to subserve a better purpose than the sponge gold, as there is a very great difficulty in introducing this *kind* of prepared material. Great care should be taken, if you wish to insert more than one tooth upon two or more fangs, to have the tubes parallel to each other, as in the failure to do this there will be great difficulty in adapting the teeth properly and securing the tubes within the fangs.

I may as well say here, that in many instances, the diseased conditions of the membranes, investing the fangs are such that the secretions at the apex cannot be arrested entirely by any known treatment. To obviate any difficulty that must arise from blocking up the avenue to the escape of such secretions, I invariably use a hollow pivot with the cavity extending entirely through the base. For this peculiarity I am indebted to a member of the profession distinguished for his skill and attainments. The novelty of my mode consists in availing myself of the suggestions arising from this method to insert *more* than one tooth upon *one* or *more* fangs, without the introduction of *plate* upon any portion of the *alveolar* ridge, or *clasps around* the teeth.

The process is simple and obvious. After tubing, I adjust all the teeth as I wish them, letting the crowns touch down upon the gums, where there are no fangs—place plaster and sand around them—back them all up—making the proximal edges of the backings in each tooth to come in contact two-thirds of the length of the tooth, from the crown towards the cutting edge.

The teeth having no fangs to correspond with them in the alveolar ridge will need nothing but the backings to support them. Then I solder the teeth having pivots to them, to their bases in the usual way, at the same time I unite the edges of each backing of all the teeth in the same manner. Thus affording an ample support laterally to all the teeth having no pivots to them to those that have.

In this way six or even eight of the anterior teeth may be very successfully inserted upon barely two good fangs. Three fangs would be preferable for the insertion of more than six teeth, though that number is not absolutely indispensable to the success of the operation. I like to have if I can one good fang for every two teeth inserted.

I have frequently inserted six on two fangs, and in every case, I have pleased the patient and myself too. I have never inserted any beyond the bicuspids in this way as I apprehend for the purposes of mastication they could be of little service without a plate extending over a portion of the alveolar ridge and palate.

If you have fangs located rightly, and you wish to insert six or more teeth, the judgment of the operator must determine whether it is best to construct them in one piece or in segments. It requires more skill to insert two teeth upon one fang than any other number upon two or more fangs, as the great difficulty is to prevent the teeth from rotating when there is but one pivot.

This result I obviate when I have a good sized sound fang by making a tube with a square interior, with a cylindrical exterior—the pivot of course must correspond with the interior form of the tube. If the fang is small, accuracy of fit and depth of filing in the end of the fang, with a file of small diametor is the only remedy. Square tubes are only necessary when two teeth are required to be supported by one fang.

There is another advantage arising from uniting the teeth laterally, although this is merely incidental. It sometimes happens that the teeth around which clasps have been placed are lost in a short time, and the patient is unwilling or unable to have the fangs extracted, and wait the slow process of the shrinking of the soft tissues and the absorption and filling up of the sockets of the teeth before a full set can be inserted. All that is necessary to be done, or rather all the patient will permit you to do, is to take the old teeth and solder the requisite number to them in the manner heretofore described.

It may be asked, how will you prevent the backing from showing through the approximal edges of the teeth? I answer but one way that I know of—and that is to have the teeth so close together that there will be no very perceptible space between them.

The advantages arising from this method of inserting teeth need scarcely be suggested to any professional man, as even the patients themselves can at once appreciate the benefits arising from it at a glance. In this mode you attain a result aimed at a long time, of getting rid of a huge plate in the mouth, with its attendant evils of gold clasps, impaired taste, imperfect and unnatural enunciation, and certain destruction in a short time of sound teeth, by the use of clasps and of their unsightly exhibition. The teeth will be more securely retained than by any other method, if the operations are performed as they should and can be, and I am sure all will be pleased with the result when accomplished.

In the suggestions I have given, my principal object has been to make myself understood. If I have done this I am gratified, and if the mode of practice I have detailed to you is of any service to the profession, I shall rejoice to think I have contributed a mite to extend its usefulness.

DECATUR P. GREGG.

GREENSBORO', N. C., *Dec. 1st,* 1857.

